# Structural (at 100 K) and DFT studies of 2′-nitro­flavone

**DOI:** 10.1107/S2056989020010713

**Published:** 2020-08-07

**Authors:** Evgenii Oskolkov, Tatiana Kornilova, Preciosa America Chavez, John P. Tillotson, Tatiana V. Timofeeva

**Affiliations:** a Department of Chemistry, New Mexico Highlands University, Las Vegas, New Mexico, 87701, USA; b School of Chemistry and Biochemistry, Georgia Institute of Technology, Atlanta, Georgia, 30332, USA

**Keywords:** crystal structure, nitro­flavone, anti-cancer agent, π-π- inter­actions, DFT calculations

## Abstract

The geometry of the title mol­ecule [systematic name: 2-(2-nitro­phen­yl)-4*H*-chromen-4-one], C_15_H_9_NO_4_, is determined by two dihedral angles formed by the mean plane of phenyl ring with the mean planes of chromone moiety and nitro group, being 50.73 (5) and 30.89 (7)°, respectively. The crystal packing is determined by π–π inter­actions and C—H⋯O contacts. The results of DFT calculations at the B3LYP/6–31G* level of theory provided an explanation of the unusually large dihedral angle between the chromone moiety and the phenyl group. The electrostatic potential map on the mol­ecular surface was calculated in order to determine the potential binding sites to receptors.

## Abstract 2

The title mol­ecule, C_15_H_9_NO_4_, is non-planar, with the unusually large dihedral angle between mean planes of phenyl ring and chromone moiety being 50.73 (5)°.

## Chemical context   

The naturally occurring group of heterocyclic compounds known as flavonoids has received considerable attention over the past 15 years. The synthesis and applications of flavones and their derivatives have been studied extensively because of their diverse pharmaceutical properties. Besides their physiological role in plants (Agati *et al.*, 2012 ▸), this class of compounds has demonstrated anti­allergic, anti­viral, anxiolytic and anti-inflammatory activities (Manthey *et al.*, 2001 ▸). Several synthetic flavonoids and their nitro derivatives, including a few halogen-substituted compounds, have been found to act as highly competitive ligands for benzodiazepine receptors, suggesting a possible use as anxiolytic drugs (Marder *et al.*, 1995 ▸). Most importantly, several nitro derivatives of flavones have been reported to possess anti­proliferative properties against human and murine cancerous cells, by the mechanism of induced apoptosis (Blank *et al.*, 2004 ▸). Moreover, some flavonoids have been found to be capable of restoring the viability of human vascular endothelial cells, thus providing both cytoprotective and cytotoxic effects on normal and cancerous cells, respectively (Ramos, 2008 ▸). The title compound, 2′-nitro­flavone, has previously been shown to effectively inhibit human and murine tumor cell activity without affecting the non-tumor cells. Induced apoptosis mol­ecular mechanisms have been studied *in vitro* for HeLa human cervix carcinoma (Cárdenas *et al.*, 2008 ▸) and *in vivo* in murine adenocarcinoma cells (Cárdenas *et al.*, 2009 ▸). Several haematological cancer cell lines were used in the cytotoxicity evaluation of the title compound, along with a culture of healthy peripheral blood mononuclear cells (PBMCs); the IC_50_ values (drug concentrations needed to induce a 50% inhibition of cell growth) after 2′-nitro­flavone treatment ranged from 1±0.5 µmol L^−1^ to 9±1.4 µmol/L for various neoplastic cells, while the healthy cells IC_50_ was found to be over 80 µmol L^−1^, effectively leaving the cells intact under the concentrations sufficient for cancerous cells (Cárdenas *et al.*, 2012 ▸). Despite the evident importance of nitro­flavone derivatives, structural studies until now have been limited to only one reported nitro­flavone-based compound (Kendi *et al.*, 1996 ▸). In this work, a combined study consisting of X-ray diffraction (XRD) structural analysis and quantum-chemical DFT calculations was carried out in order to obtain insight into the structure–property relationship, and more specifically the effect of the nitro substituent in the *ortho*-position of the phenyl moiety of a flavone.
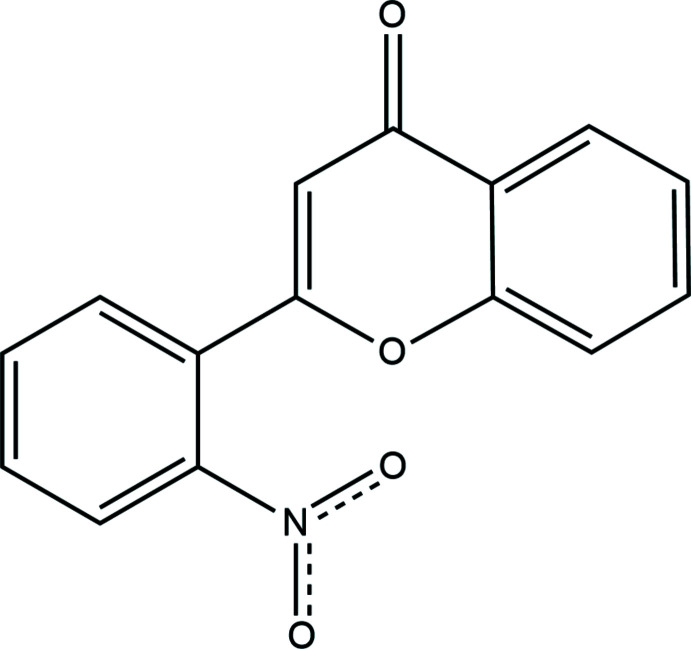


## Structural commentary   

**Figure 1:**
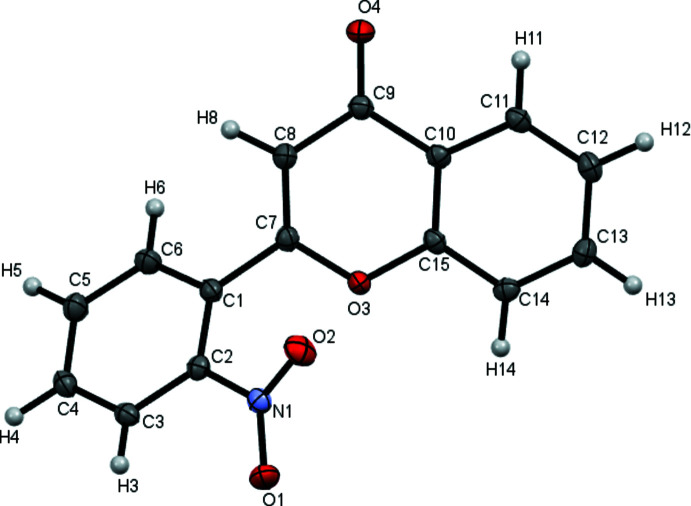
A view of the mol­ecular structure of the title compound with the atom-labeling scheme. Displacement ellipsoids are drawn at the 50% probability level.

The mol­ecular structure of 2′-nitro­flavone is presented in Fig. 1 ▸. The mean plane of the benzene ring makes dihedral angles of 50.73 (5) and 30.89 (7)° with the mean planes of the chromone moiety and the nitro group, respectively. The dihedral angle between mean planes of the chromone and benzene groups is unusually large when compared to other *ortho*-substituted flavone derivatives. For instance, the mol­ecule of 2′-meth­oxy­flavone was reported to be almost planar, with a dihedral angle of 2.9° (Wallet *et al.*, 1990 ▸). Even in the flavonoid with a bulky carbazole substituent in the same position, this dihedral angle is only 29.2° (Zheng, 2018 ▸). The length of the single bond between the chromone and benzene moieties is 1.469 (2) Å, indicating some π–π conjugation. The unusually large dihedral angle in the title mol­ecule can be attributed to the steric tension between the nitro group and the chromone oxygen atom, whereas in the carbazole derivative this substituent is twisted far enough from the plane of the benzene ring to avoid it coming into close proximity with the flavone core.

## Supra­molecular features   

**Figure 2:**
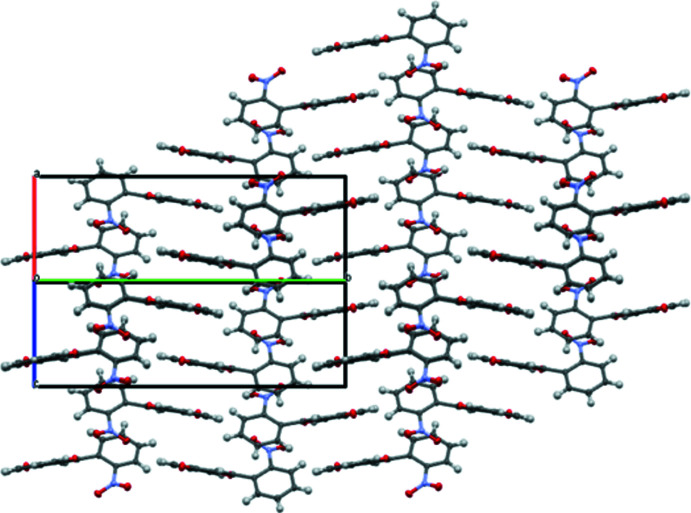
Parquet-like mol­ecular packing in the title structure.

**Figure 3:**
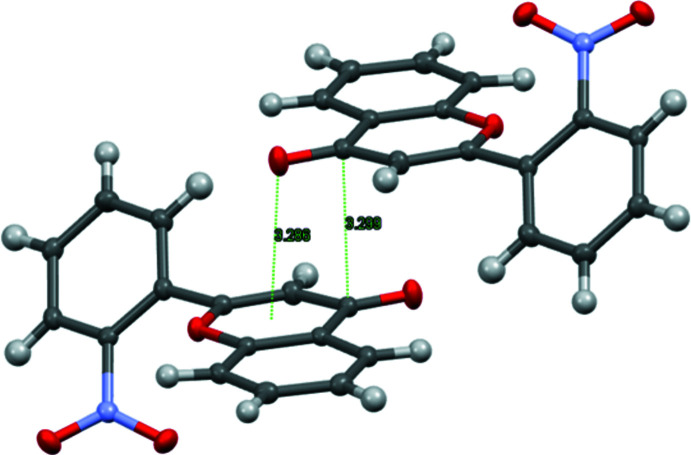
Short inter­molecular C⋯C and C—O⋯π contacts in the crystal of the title compound.

In the crystal, the title mol­ecules form a parquet-like structure, with alternating layers of coplanar chromone backbones (Fig. 2 ▸). The presence of π–π inter­actions in the crystal packing can be suggested from the short inter­molecular distance of 3.299 (4) Å between the overlapping C9 atoms from opposing mol­ecules. Moreover, a short contact of 3.286 (3) Å between the carbonyl oxygen atom and the centroid of the opposing heterocyclic ring is found, which suggests an inter­action of the oxygen atom with the π-system (Fig. 3 ▸). Such an inter­molecular inter­action was found in the crystal structure of chiral amino alcohol with a penta­fluoro­phenyl group (Korenaga *et al.*, 2003 ▸). Two short C—H⋯O contacts also occur, indicating at additional structural stability (Table 1 ▸).

## Database survey   

A search of the Cambridge Crystallographic Database (CSD version 5.40, update of September 19; Groom *et al.*, 2016 ▸) for the title mol­ecule yielded no entries. A single nitro­flavone entry, for 2′-methyl-3′-nitro­flavone, was found (REZROD; Kendi *et al.*, 1996 ▸). A search for flavone-core mol­ecules with only an *ortho*-substituted phenyl ring returned a total of six entries, three of which correspond to the compound with a meth­oxy group in the 2′-position [KEPLAS (Wallet *et al.*, 1990 ▸), KEPLAS01 (McKendall *et al.*, 2008 ▸), KEPLAS02 (Zia *et al.*., 2020 ▸)]; more specifically, one of the entries represents a structure of a possible polymorph, while the other two correspond to the same form. The other three correspond to carbazole (XIJVAQ; Zheng, 2018 ▸), hy­droxy (YUDWEZ; Seetharaman & Rajan, 1995 ▸) and ethyl glycolate (PIGXUB; Goyal *et al.*, 2018 ▸) substituents. Most of these mol­ecules exhibit only slight deviations from planarity, with the exception of carbazole-substituted mol­ecule.

## DFT calculations   

In an attempt to get further insight into structure and properties of the title mol­ecule (I), a DFT study was carried out at the B3LYP/6-31G* level of theory with *GAUSSIAN 16* (Frisch *et al.*, 2016 ▸) software. The geometry of the ground state was optimized, using the XRD data as a starting point. The optimized geometry was confirmed to be the minimum by vibrational frequency analysis. Two previously described flavonoids, with meth­oxy (II) and carbazole (III) substituents in the 2′-position, were also optimized and compared with the XRD data. Selected geometrical parameters are presented in Tables 2 ▸–4 ▸ ▸.

The calculated parameters are in satisfactory agreement with those obtained experimentally. The range of calculated dihedral angles between the moieties comprising the flavone core is narrower than that observed in the crystal structures, but still demonstrates the same trend with the title compound having the largest angle.

**Figure 4:**
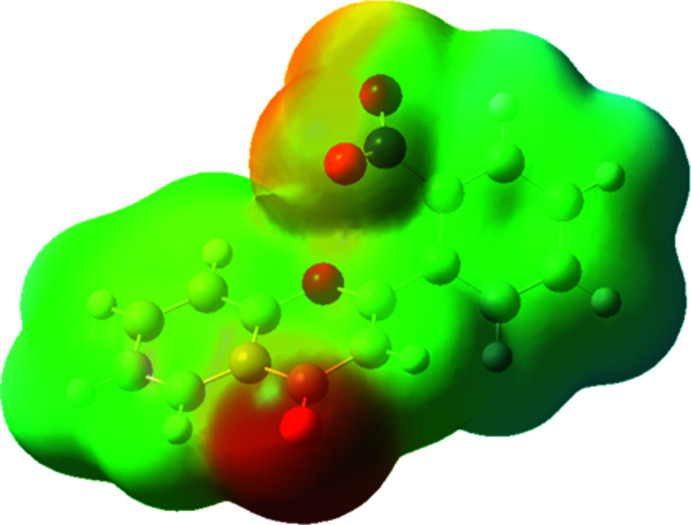
Electrostatic potential on the van der Waals surface of the title compound.

Considering the importance of the biological functions of the title compound, including its ability to competitively bind to benzodiazepine receptors, the electrostatic potential on the van der Waals surface was calculated (Fig. 4 ▸). While initially we had expected the nitro group to be the negative charge concentration site, it turned out to be the oxygen of the chromone moiety. We speculate that it could be the binding part in this mol­ecule’s inter­action with benzodiazepine receptors.

## Synthesis and crystallization   

The synthesis of the title compound was performed as described in the literature (Barros & Silva, 2006 ▸). The obtained product was recrystallized by slow evaporation from ethanol solution.

## Refinement   

Crystal data, data collection and structure refinement details are summarized in Table 5 ▸. Data collection was performed at 100 K. All hydrogen atoms were located from the difference-Fourier map and freely refined.

## Supplementary Material

Crystal structure: contains datablock(s) I. DOI: 10.1107/S2056989020010713/yk2138sup1.cif

Structure factors: contains datablock(s) I. DOI: 10.1107/S2056989020010713/yk2138Isup3.hkl

Supporting information file. DOI: 10.1107/S2056989020010713/yk2138Isup3.cml

CCDC reference: 2021153

Additional supporting information:  crystallographic information; 3D view; checkCIF report

## Figures and Tables

**Table 1:** Hydrogen-bond geometry (Å, °)

*D*—H⋯*A*	*D*—H	H⋯*A*	*D*⋯*A*	*D*—H⋯*A*
C3—H3⋯O4^i^	0.933 (17)	2.675 (16)	3.198 (3)	116.1 (12)
C4—H4⋯O4^i^	0.971 (16)	2.446 (16)	3.109 (3)	125.3 (12)

Symmetry code: (i) 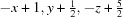.

**Table 2:** Experimental (XRD) and calculated (DFT) dihedral angles (°) between the phenyl and chromone moieties in the title mol­ecule (I), the 2′-meth­oxy derivative (II) and the 2′-carbazole derivative (III)

	XRD	DFT
(I)	50.73 (5)	47.56
(II)	2.89 (7)	22.16
(III)	29.21 (6)	40.13

**Table 3:** Experimental (XRD) and calculated (DFT) lengths of single bonds (Å) between the phenyl and chromone moieties in the title mol­ecule (I), the 2′-meth­oxy derivative (II) and the 2′-carbazole derivative (III)

	XRD	DFT
(I)	1.469 (2)	1.482
(II)	1.475 (4)	1.477
(III)	1.478 (2)	1.481

**Table 4:** Experimental (XRD) and calculated (DFT) dihedral/torsion angles (°) between the phenyl group and the substituent in the 2′-position in the title mol­ecule (I), the meth­oxy derivative (II) and the carbazole derivative (III)

	XRD	DFT
(I)	30.89 (7)	31.83
(II)	174.3 (2)	176.78
(III)	69.95 (9)	66.40

**Table 5:** Experimental details

Crystal data	
Chemical formula	C_15_H_9_NO_4_
*M*_r_	267.23
Crystal system, space group	Monoclinic, *P*2_1_/*c*
Temperature (K)	100
*a*, *b*, *c* (Å)	8.079 (7), 20.134 (17), 7.915 (7)
β (°)	116.647 (18)
*V* (Å^3^)	1150.6 (16)
*Z*	4
Radiation type	Mo *K*α
μ (mm^−1^)	0.11
Crystal size (mm)	0.35 × 0.28 × 0.25

Data collection	
Diffractometer	Bruker APEXII CCD
No. of measured, independent and observed [*I* > 2σ(*I*)] reflections	9944, 2491, 2248
*R*_int_	0.034
(sin θ/λ)_max_ (Å^−1^)	0.650

Refinement	
*R*[*F* ^2^ > 2σ(*F* ^2^)], *wR*(*F* ^2^), *S*	0.035, 0.093, 1.05
No. of reflections	2491
No. of parameters	217
H-atom treatment	All H-atom parameters refined
Δρ_max_, Δρ_min_ (e Å^−3^)	0.30, −0.23

Computer programs: *APEX3* and *SAINT* (Bruker, 2016 ▸), *SHELXT2017/1* (Sheldrick, 2015*a* ▸), *SHELXL2017/1* (Sheldrick, 2015*b* ▸) and *Mercury* (Macrae *et al.*, 2020 ▸).

## supplementary crystallographic information

### Crystal data

**Table:**

C_15_H_9_NO_4_	*F*(000) = 552
*M**_r_* = 267.23	*D*_x_ = 1.543 Mg m^−^^3^
Monoclinic, *P*2_1_/*c*	Mo *K*α radiation, λ = 0.71073 Å
*a* = 8.079 (7) Å	Cell parameters from 1326 reflections
*b* = 20.134 (17) Å	θ = 3.1–32.1°
*c* = 7.915 (7) Å	µ = 0.11 mm^−^^1^
β = 116.647 (18)°	*T* = 100 K
*V* = 1150.6 (16) Å^3^	Block-shaped, white
*Z* = 4	0.35 × 0.28 × 0.25 mm

### Data collection

**Table:**

Bruker APEXII CCD diffractometer	*R*_int_ = 0.034
φ and ω scans	θ_max_ = 27.5°, θ_min_ = 2.0°
9944 measured reflections	*h* = −10→6
2491 independent reflections	*k* = −16→26
2248 reflections with *I* > 2σ(*I*)	*l* = −10→8

### Refinement

**Table:**

Refinement on *F*^2^	Primary atom site location: iterative
Least-squares matrix: full	Hydrogen site location: difference Fourier map
*R*[*F*^2^ > 2σ(*F*^2^)] = 0.035	All H-atom parameters refined
*wR*(*F*^2^) = 0.093	*w* = 1/[σ^2^(*F*_o_^2^) + (0.0401*P*)^2^ + 0.551*P*] where *P* = (*F*_o_^2^ + 2*F*_c_^2^)/3
*S* = 1.05	(Δ/σ)_max_ = 0.001
2491 reflections	Δρ_max_ = 0.30 e Å^−^^3^
217 parameters	Δρ_min_ = −0.23 e Å^−^^3^
0 restraints	

### Special details

**Table:**

Geometry. All esds (except the esd in the dihedral angle between two l.s. planes) are estimated using the full covariance matrix. The cell esds are taken into account individually in the estimation of esds in distances, angles and torsion angles; correlations between esds in cell parameters are only used when they are defined by crystal symmetry. An approximate (isotropic) treatment of cell esds is used for estimating esds involving l.s. planes.

### Fractional atomic coordinates and isotropic or equivalent isotropic displacement parameters (Å^2^)

**Table:**

	*x*	*y*	*z*	*U*_iso_*/*U*_eq_	
O3	0.22049 (12)	0.13047 (4)	0.89731 (12)	0.0160 (2)	
O4	0.48862 (13)	−0.02543 (4)	1.23925 (13)	0.0229 (2)	
O1	0.04077 (13)	0.29953 (4)	0.98796 (14)	0.0230 (2)	
O2	0.11312 (14)	0.20433 (4)	1.12606 (14)	0.0243 (2)	
C7	0.37958 (17)	0.13828 (6)	1.06058 (17)	0.0154 (2)	
C10	0.22802 (17)	0.01385 (6)	0.96633 (16)	0.0146 (2)	
C9	0.40481 (17)	0.02123 (6)	1.13800 (17)	0.0160 (2)	
C15	0.14347 (17)	0.06830 (5)	0.85277 (17)	0.0144 (2)	
C14	−0.02257 (17)	0.06319 (6)	0.68804 (17)	0.0163 (2)	
C2	0.33977 (17)	0.26206 (6)	1.08494 (16)	0.0148 (2)	
C13	−0.10846 (18)	0.00246 (6)	0.63791 (17)	0.0182 (3)	
C1	0.44677 (17)	0.20715 (6)	1.09092 (16)	0.0154 (2)	
N1	0.15066 (15)	0.25441 (5)	1.06335 (14)	0.0164 (2)	
C8	0.47167 (18)	0.08861 (6)	1.17707 (17)	0.0175 (3)	
C11	0.13820 (18)	−0.04727 (6)	0.91170 (17)	0.0168 (3)	
C6	0.62821 (18)	0.21870 (6)	1.12309 (18)	0.0194 (3)	
C3	0.40847 (18)	0.32564 (6)	1.10911 (17)	0.0165 (3)	
C5	0.69942 (19)	0.28224 (6)	1.14845 (19)	0.0204 (3)	
C4	0.58971 (18)	0.33551 (6)	1.14135 (17)	0.0185 (3)	
C12	−0.02751 (18)	−0.05292 (6)	0.75091 (18)	0.0186 (3)	
H13	−0.221 (2)	−0.0009 (7)	0.527 (2)	0.019 (4)*	
H11	0.196 (2)	−0.0854 (7)	0.994 (2)	0.020 (4)*	
H8	0.585 (2)	0.0986 (8)	1.292 (2)	0.024 (4)*	
H14	−0.071 (2)	0.1017 (8)	0.610 (2)	0.020 (4)*	
H3	0.334 (2)	0.3611 (8)	1.110 (2)	0.025 (4)*	
H4	0.638 (2)	0.3804 (8)	1.162 (2)	0.025 (4)*	
H5	0.825 (2)	0.2894 (7)	1.172 (2)	0.022 (4)*	
H12	−0.090 (2)	−0.0948 (8)	0.716 (2)	0.024 (4)*	
H6	0.706 (2)	0.1816 (8)	1.128 (2)	0.027 (4)*	

### Atomic displacement parameters (Å^2^)

**Table:**

	*U*^11^	*U*^22^	*U*^33^	*U*^12^	*U*^13^	*U*^23^
O3	0.0160 (5)	0.0119 (4)	0.0168 (4)	−0.0011 (3)	0.0043 (4)	−0.0001 (3)
O4	0.0205 (5)	0.0164 (4)	0.0244 (5)	0.0012 (3)	0.0034 (4)	0.0050 (3)
O1	0.0174 (5)	0.0199 (4)	0.0291 (5)	0.0041 (3)	0.0083 (4)	0.0014 (4)
O2	0.0265 (5)	0.0205 (4)	0.0309 (5)	−0.0026 (4)	0.0175 (5)	0.0039 (4)
C7	0.0149 (6)	0.0148 (5)	0.0168 (5)	−0.0012 (4)	0.0073 (5)	−0.0026 (4)
C10	0.0151 (6)	0.0138 (5)	0.0166 (5)	0.0006 (4)	0.0086 (5)	−0.0012 (4)
C9	0.0158 (6)	0.0151 (5)	0.0173 (5)	0.0008 (4)	0.0077 (5)	0.0007 (4)
C15	0.0161 (6)	0.0125 (5)	0.0169 (5)	−0.0010 (4)	0.0094 (5)	−0.0021 (4)
C14	0.0164 (6)	0.0163 (5)	0.0164 (5)	0.0021 (4)	0.0076 (5)	0.0005 (4)
C2	0.0150 (6)	0.0162 (5)	0.0132 (5)	−0.0006 (4)	0.0062 (5)	−0.0005 (4)
C13	0.0158 (6)	0.0215 (6)	0.0159 (5)	−0.0023 (5)	0.0059 (5)	−0.0034 (4)
C1	0.0173 (6)	0.0129 (5)	0.0146 (5)	−0.0005 (4)	0.0059 (5)	−0.0006 (4)
N1	0.0174 (5)	0.0156 (5)	0.0162 (5)	0.0000 (4)	0.0076 (4)	−0.0020 (4)
C8	0.0165 (6)	0.0160 (6)	0.0169 (5)	−0.0005 (4)	0.0046 (5)	−0.0014 (4)
C11	0.0191 (6)	0.0139 (5)	0.0195 (6)	0.0006 (4)	0.0106 (5)	−0.0009 (4)
C6	0.0182 (7)	0.0156 (6)	0.0236 (6)	0.0009 (5)	0.0087 (5)	−0.0017 (5)
C3	0.0201 (6)	0.0130 (5)	0.0161 (5)	0.0010 (4)	0.0078 (5)	0.0000 (4)
C5	0.0163 (7)	0.0195 (6)	0.0252 (6)	−0.0023 (5)	0.0092 (5)	−0.0012 (5)
C4	0.0211 (7)	0.0142 (5)	0.0201 (6)	−0.0032 (5)	0.0091 (5)	−0.0012 (4)
C12	0.0215 (7)	0.0157 (5)	0.0209 (6)	−0.0037 (5)	0.0115 (5)	−0.0043 (4)

### Geometric parameters (Å, º)

**Table:**

O3—C7	1.3626 (17)	C2—C3	1.3741 (19)
O3—C15	1.3717 (16)	C13—C12	1.3950 (19)
O4—C9	1.2233 (16)	C1—C6	1.390 (2)
O1—N1	1.2224 (15)	C11—C12	1.377 (2)
O2—N1	1.2208 (15)	C6—C5	1.380 (2)
C7—C1	1.4690 (19)	C3—C4	1.383 (2)
C7—C8	1.3373 (18)	C5—C4	1.3765 (19)
C10—C9	1.4722 (19)	C14—H14	0.958 (15)
C10—C15	1.3877 (18)	C13—H13	0.944 (16)
C10—C11	1.3950 (19)	C11—H11	0.979 (15)
C9—C8	1.4415 (19)	C6—H6	0.964 (17)
C15—C14	1.3932 (19)	C3—H3	0.933 (17)
C14—C13	1.3736 (19)	C5—H5	0.953 (17)
C2—C1	1.3911 (18)	C4—H4	0.971 (16)
C2—N1	1.467 (2)	C12—H12	0.957 (16)
			
C7—O3—C15	118.38 (9)	C12—C11—C10	120.95 (12)
O3—C7—C1	112.72 (10)	C5—C6—C1	121.21 (12)
C8—C7—O3	124.33 (11)	C2—C3—C4	119.18 (11)
C8—C7—C1	122.86 (12)	C4—C5—C6	119.85 (14)
C15—C10—C9	120.66 (11)	C5—C4—C3	120.30 (12)
C15—C10—C11	117.36 (12)	C11—C12—C13	120.55 (12)
C11—C10—C9	121.98 (11)	C13—C14—H14	121.7 (9)
O4—C9—C10	123.36 (12)	C15—C14—H14	119.2 (9)
O4—C9—C8	122.56 (13)	C14—C13—H13	119.0 (9)
C8—C9—C10	114.08 (10)	C12—C13—H13	121.3 (9)
O3—C15—C10	121.31 (12)	C7—C8—H8	119.2 (9)
O3—C15—C14	116.32 (10)	C9—C8—H8	119.7 (9)
C10—C15—C14	122.37 (11)	C12—C11—H11	121.7 (9)
C13—C14—C15	119.06 (11)	C10—C11—H11	117.3 (9)
C1—C2—N1	121.28 (11)	C5—C6—H6	119.4 (10)
C3—C2—C1	121.98 (13)	C1—C6—H6	119.4 (10)
C3—C2—N1	116.64 (11)	C2—C3—H3	119.6 (10)
C14—C13—C12	119.70 (13)	C4—C3—H3	121.1 (10)
C2—C1—C7	124.49 (12)	C4—C5—H5	119.8 (9)
C6—C1—C7	118.02 (11)	C6—C5—H5	120.4 (9)
C6—C1—C2	117.47 (11)	C5—C4—H4	120.9 (10)
O1—N1—C2	117.98 (11)	C3—C4—H4	118.8 (10)
O2—N1—O1	123.63 (12)	C11—C12—H12	120.5 (9)
O2—N1—C2	118.35 (10)	C13—C12—H12	118.9 (9)
C7—C8—C9	121.12 (12)		
			
O3—C7—C1—C2	52.15 (16)	C2—C1—C6—C5	0.11 (19)
O3—C7—C1—C6	−126.13 (13)	C2—C3—C4—C5	0.21 (18)
O3—C7—C8—C9	−1.1 (2)	C1—C7—C8—C9	−177.40 (11)
O3—C15—C14—C13	−178.69 (11)	C1—C2—N1—O1	−152.23 (12)
O4—C9—C8—C7	178.00 (12)	C1—C2—N1—O2	29.74 (16)
C7—O3—C15—C10	−2.47 (17)	C1—C2—C3—C4	−0.34 (18)
C7—O3—C15—C14	177.77 (10)	C1—C6—C5—C4	−0.2 (2)
C7—C1—C6—C5	178.51 (12)	N1—C2—C1—C7	5.50 (17)
C10—C9—C8—C7	−1.92 (18)	N1—C2—C1—C6	−176.22 (10)
C10—C15—C14—C13	1.55 (19)	N1—C2—C3—C4	176.21 (10)
C10—C11—C12—C13	0.61 (19)	C8—C7—C1—C2	−131.15 (14)
C9—C10—C15—O3	−0.57 (18)	C8—C7—C1—C6	50.57 (18)
C9—C10—C15—C14	179.18 (11)	C11—C10—C9—O4	3.2 (2)
C9—C10—C11—C12	179.73 (11)	C11—C10—C9—C8	−176.84 (11)
C15—O3—C7—C1	−179.96 (10)	C11—C10—C15—O3	178.98 (11)
C15—O3—C7—C8	3.39 (18)	C11—C10—C15—C14	−1.27 (18)
C15—C10—C9—O4	−177.23 (12)	C6—C5—C4—C3	0.07 (19)
C15—C10—C9—C8	2.69 (17)	C3—C2—C1—C7	−178.11 (11)
C15—C10—C11—C12	0.18 (18)	C3—C2—C1—C6	0.18 (18)
C15—C14—C13—C12	−0.71 (19)	C3—C2—N1—O1	31.19 (15)
C14—C13—C12—C11	−0.33 (19)	C3—C2—N1—O2	−146.84 (12)

### Hydrogen-bond geometry (Å, º)

**Table:**

*D*—H···*A*	*D*—H	H···*A*	*D*···*A*	*D*—H···*A*
C3—H3···O4^i^	0.933 (17)	2.675 (16)	3.198 (3)	116.1 (12)
C4—H4···O4^i^	0.971 (16)	2.446 (16)	3.109 (3)	125.3 (12)

Symmetry code: (i) −*x*+1, *y*+1/2, −*z*+5/2.

## References

[bb1] Agati, G., Azzarello, E., Pollastri, S. & Tattini, M. (2012). *Plant Sci.* **196**, 67–76. 10.1016/j.plantsci.2012.07.014 23017900

[bb2] Barros, A. I. R. N. A. & Silva, A. M. S. (2006). *Monatsh. Chem.* **137**, 1505–1528.

[bb3] Blank, V. C., Poli, C., Marder, M. & Roguin, L. P. (2004). *Bioorg. Med. Chem. Lett.* **14**, 133–136. 10.1016/j.bmcl.2003.10.029 14684314

[bb4] Bruker (2016). *APEX3* and *SAINT*. Bruker AXS Inc., Madison, Wisconsin, USA.

[bb5] Cárdenas, M. G., Blank, V. C., Marder, M. & Roguin, L. P. (2008). *Cancer Lett.* **268**, 146–157. 10.1016/j.canlet.2008.03.062 18485587

[bb6] Cárdenas, M. G., Blank, V. C., Marder, M. N. & Roguin, L. P. (2012). *Anticancer Drugs*, **23**, 815–826. 10.1097/CAD.0b013e328353f947 22555195

[bb7] Cárdenas, M. G., Zotta, E., Marder, M. & Roguin, L. P. (2009). *Int. J. Cancer*, **125**, 222–228. 10.1002/ijc.24361 19358271

[bb8] Frisch, M. J., Trucks, G. W., Schlegel, H. B., Scuseria, G. E., Robb, M. A., Cheeseman, J. R., Montgomery, J. A. Jr, Vreven, T., Kudin, K. N., Burant, J. C., Millam, J. M., Iyengar, S. S., Tomasi, J., Barone, V., Mennucci, B., Cossi, M., Scalmani, G., Rega, N., Petersson, G. A., Nakatsuji, H., Hada, M., Ehara, M., Toyota, K., Fukuda, R., Hasegawa, J., Ishida, M., Nakajima, T., Honda, Y., Kitao, O., Nakai, H., Klene, M., Li, X., Knox, J. E., Hratchian, H. P., Cross, J. B., Bakken, V., Adamo, C., Jaramillo, J., Gomperts, R., Stratmann, R. E., Yazyev, O., Austin, A. J., Cammi, R., Pomelli, C., Ochterski, J. W., Ayala, P. Y., Morokuma, K., Voth, G. A., Salvador, P., Dannenberg, J. J., Zakrzewski, V. G., Dapprich, S., Daniels, A. D., Strain, M. C., Farkas, O., Malick, D. K., Rabuck, A. D., Raghavachari, K., Foresman, J. B., Ortiz, J. V., Cui, Q., Baboul, A. G., Clifford, S., Cioslowski, J., Stefanov, B. B., Liu, G., Liashenko, A., Piskorz, P., Komaromi, I., Martin, R. L., Fox, D. J., Keith, T., Al-Laham, M. A., Peng, C. Y., Nanayakkara, A., Challacombe, M., Gill, P. M. W., Johnson, B., Chen, W., Wong, M. W., Gonzalez, C. & Pople, J. A. (2016). *GAUSSIAN016.* Rev. C.01. Gaussian Inc., Wallingford, CT, USA. http://www.gaussian.com.

[bb9] Goyal, N., Do, C., Donahue, J. P., Mague, J. T. & Foroozesh, M. (2018). *IUCrData*, **3**, x180993. 10.1107/S2414314618009938 PMC6457468 30984887

[bb10] Groom, C. R., Bruno, I. J., Lightfoot, M. P. & Ward, S. C. (2016). *Acta Cryst.* B**72**, 171–179. 10.1107/S2052520616003954 PMC4822653 27048719

[bb11] Kendi, E., Özbey, S., Tunçbilek, M. & Ertan, R. (1996). *Cryst. Res. Technol.* **31**, 611–615.

[bb12] Korenaga, T., Tanaka, H., Ema, T. & Sakai, T. (2003). *J. Fluor. Chem.* **122**, 201–205.

[bb13] Macrae, C. F., Sovago, I., Cottrell, S. J., Galek, P. T. A., McCabe, P., Pidcock, E., Platings, M., Shields, G. P., Stevens, J. S., Towler, M. & Wood, P. A. (2020). *J. Appl. Cryst.* **53**, 226–235. 10.1107/S1600576719014092 PMC6998782 32047413

[bb14] Manthey, J., Grohmann, K. & Guthrie, N. (2001). *Curr. Med. Chem.* **8**, 135–153. 10.2174/0929867013373723 11172671

[bb15] Marder, M., Viola, H., Wasowski, C., Wolfman, C., Waterman, P. G., Medina, J. H. & Paladini, A. C. (1995). *Bioorg. Med. Chem. Lett.* **5**, 2717–2720.

[bb16] McKendall, M., Smith, T., Anh, K., Ellis, J., McGee, T., Foroozesh, M., Zhu, N. & Stevens, C. L. K. (2008). *J. Chem. Crystallogr.* **38**, 231–237.

[bb17] Ramos, S. (2008). *Mol. Nutr. Food Res.* **52**, 507–526.

[bb18] Seetharaman, J. & Rajan, S. S. (1995). *Z. Kristallogr.* **210**, 104–106.

[bb19] Sheldrick, G. M. (2015*a*). *Acta Cryst.* A**71**, 3–8.

[bb20] Sheldrick, G. M. (2015*b*). *Acta Cryst.* C**71**, 3–8.

[bb21] Wallet, J.-C., Gaydou, E. M., Jaud, J. & Baldy, A. (1990). *Acta Cryst.* C**46**, 1536–1540.

[bb22] Zheng, Z. K. (2018). CSD Communication (refcode XIJVAQ). CCDC, Cambridge, England.

[bb23] Zia, M., Khalid, M., Hameed, S., Irran, E. & Naseer, M. M. (2020). *J. Mol. Struct.* 1207 Article 127811.

